# Role Theory: A Framework to Explore Health Professional Perceptions of Expanding Rural Community Pharmacists’ Role

**DOI:** 10.3390/pharmacy8030161

**Published:** 2020-09-02

**Authors:** Selina Taylor, Alice Cairns, Beverley Glass

**Affiliations:** 1 Centre for Rural and Remote Health, James Cook University, Mount Isa 4825, QLD, Australia; 2 Centre for Rural and Remote Health, James Cook University, Weipa 4874, QLD, Australia; alice.cairns@jcu.edu.au; 3 Pharmacy Department, James Cook University, Townsville 4825, QLD, Australia; beverley.glass@jcu.edu.au

**Keywords:** pharmacy practice, model of care, expanded practice, extended practice, full scope of practice

## Abstract

Pharmaceutical care is a concept which has moved the pharmacy profession from their primary focus on the product to optimising drug therapy for the individual patient. Expanded pharmacy practice beyond pharmaceutical care will further challenge the role perceptions that other health professionals have about pharmacists. Role theory as a philosophical perspective was used to explore rural and remote health professionals’ beliefs on pharmacists expanding their clinical role by conducting twenty-three semi-structured interviews. Five role theory categories described the data, role ambiguity, role conflict, role overload, role identity and role insufficiency. The health professionals interviewed were found to be uncertain about the boundaries between the traditional roles of the pharmacist compared to that of the expanded roles. A perceived lack of accountability by pharmacists was seen as a major contributor to role conflict, which in turn was found to impact the ability of pharmacists and other health professionals to work collaboratively. Perspectives of other health professionals on pharmacists adopting expanded practice models has highlighted significant concerns with role conflict and role identity. Acknowledging and developing clear strategies to address these concerns is essential to ensure that expanded pharmacy practice can be effectively integrated to improve access to health services and thus health outcomes for rural Australians.

## 1. Introduction

A pharmacist can be defined as a person whose job is to prepare medicines and to sell or supply them to the public in a store or in a hospital [1]. This definition, although accurate, essentially represents only the supply function performed by pharmacists. The role of the pharmacist has however evolved since the early 1900s, when pharmacists were apothecaries preparing drug products for medicinal use [2]. By the 1950s, large-scale manufacturing of most therapeutic agents shifted pharmacists’ responsibility to still include some compounding but mainly to dispensing and labelling commercial products [2]. During the mid-1960s, pharmacists assumed a more patient-orientated role and the concept of clinical pharmacy developed [2]. This signalled the start of a rapid transition period of expansion and integration of professional functions and closer interaction with doctors and other health professionals [2]. A pharmaceutical care model was developed in the 1990s to highlight the pharmacists’ role in “the responsible provision of drug therapy for the purpose of achieving definite outcomes that improve a patient’s quality of life” [3]. In the early 2000s, pharmacists were finally recognised as drug experts who would collaborate with patients, doctors and other health professionals to optimize medication management to achieve positive health outcomes [2].

Twenty years later, the pharmacy profession has witnessed major developments in health service delivery, especially in metropolitan cities, although these advancements are often not apparent in rural and remote areas [4]. For those living rurally, complex health profiles, shorter life expectancies, higher rates of disease and injury, and poorer access to and use of health services is accepted as the norm [5]. Rural community pharmacists have been identified as highly skilled health professionals who are ideally placed to address these shortcomings by providing a variety of expanded services, including disease screening and management, vaccinations and health promotion [6]. For rural pharmacists working in this challenging environment, their role is often poorly defined with ambiguous boundaries to their recognised scope of practice [7].

Pharmacists are now well recognised as medication experts with a primary role of ensuring the safe, effective and judicious supply of medicines [8]. However, they are increasingly expanding their role by developing skills and knowledge to provide expanded services [9]. Community pharmacists are becoming more widely accepted in non-dispensing roles in settings including multidisciplinary clinical teams, general practice, aged care and Indigenous Health Services [8]. As the role of the pharmacist continues to evolve, it is expected that pharmacists will be embedded into all settings where medicines are used or provided in future practice [8]. Various prescribing models including, adapting a prescription and initiating/managing drug therapy have been implemented internationally [2]. In addition, rural community pharmacists worldwide have been trialling and providing expanded services, which include point-of-care testing for chronic disease management (e.g., HbA1c/cholesterol/spirometry), infectious diseases (e.g., malaria/HIV), disease screening, immunisation and mental health management [10].

This expansion of the role of the pharmacist has resulted in various terms being put forward to describe these new roles, including expanded pharmacy practice, pharmacists’ full scope of practice, extended practice and a rural scope of practice for pharmacists, all implying the provision of services in addition to medication management [10,11,12,13,14]. These terms are often used interchangeably and with little clarity surrounding their definition [10,11,12,13,14]. The terms are distinct from advanced practice which describes an acquired higher level of expertise to achieve a higher performance level [13]. This highlights a lack of understanding of both the expanded roles and terminology used by pharmacists, other health professionals and the wider community.

### Role Theory

Role theory is defined by Conway as “a collection of concepts and a variety of hypothetical formulations that predict how actors will perform in a given role or under what circumstances certain types of behaviours can be expected” [15]. Role theory can also serve as a conceptual framework, which can be used to relate the properties of an organisation or an individual [16]. A description of behaviours, characteristics, norms and values of a person or position in the context of role theory can provide a valuable framework to examine role perceptions [17]. This makes role theory ideal to provide a conceptual framework to explore the perceptions of the role of the pharmacist in expanded practice.

The aim of this study is therefore to explore rural and remote health professionals’ (doctors, nurses and allied health) beliefs of rural community pharmacists’ role in expanded pharmacy practice.

## 2. Data and Methods

### 2.1. Study Design

An ethnographic lens of rural culture was applied to this descriptive qualitative study [18]. Rural health professionals were interviewed using an in-depth semi-structured interview process.

### 2.2. Participants, Setting and Recruitment

During July–September 2019, rural Australian health professionals were invited through various rural health networks after completing a questionnaire to nominate to be interviewed [19]. To be eligible for inclusion in the study, these health professionals needed to be working in a rural or remote location as defined by the geographical classification system Modified Monash Model [20], i.e., working in large, medium and small rural towns (MM3-5) or remote and very remote communities (MM6-7) [20].

### 2.3. Procedure and Semi-Structured Interview

All health professionals who expressed interest in participating in an interview were contacted by email; provided with an information sheet; and if agreeable to the interview, returned written informed consent. Interviews were audio recorded and de-identified in the transcription process. Demographic data including age, gender, occupation and postcodes were collected. The schedule of interview questions was informed by previous research in the area and review of the literature (Appendix A).

### 2.4. Data Analysis

All interviews were transcribed verbatim, coded and categorized into emerging themes. Objectivity, assumed knowledge and bias were minimized, and five participants were engaged in a member checking process to ensure that their code/theme interpretations were an accurate representation of their voice. After 15 interviews, data saturation was achieved; however, the remaining eight participants who had volunteered to participate were included in the interview and analysis process to ensure that no emergence of new linkages or themes occurred.

The initial conventional content analysis of ten transcripts and field notes resulted in the creation of an initial code list. A coding manual containing the initial codes was then developed, and those codes that were conceptually related were combined into categories using an ethnographic technique of domain analysis [18]. Analysis was initially performed manually on paper, followed by a refined analysis, with the assistance of software program NVivo 12 [21], using a hybrid approach of inductive and deductive coding [22].

A simplified role theory framework derived from Hardy and Conway 1988 has been applied to the analysis [23]. The framework describes role constructs that are related to a role episode (cycle of role sending, a response by the focal person and the effects of that response on the role senders) [16]. Five role constructs derived from Hardy and Conway 1988 were found in the analysis and termed as themes (Table 1) [23].

### 2.5. Ethics Approval

James Cook University Human Research Ethics Committee granted ethical approval (H7845).

## 3. Results

Twenty-three interviews were conducted face-to-face (7), via video conference (2) and via telephone (14). The interview duration ranged from 17 to 50 min, with an average duration of 29 min.

The health professionals interviewed were located in Queensland (17), New South Wales (1), Victoria (2), Northern Territory (1) and South Australia (2). They included doctors (n = 8) specialising in general practice (7), emergency retrieval (2), women’s health, Indigenous health, and mental health and nurses (n = 4) specialising in midwifery (3), chronic disease, remote practice, and emergency retrieval and allied health (n = 11) including occupational therapy (2), physiotherapy (2), speech pathology (2), exercise physiology (2), public health nutrition, podiatry and Indigenous health. The mean age was 42.3 years (SD = 13), with an age range from 23–59 years. The demographic data are summarised in Table 1. Direct quotations are numbered (PX-) with each health professional’s primary occupation provided.

Participants were initially asked to describe the major health concerns for their rural community. Mental health; diabetes; and cardiovascular, renal and respiratory disease were the most prevalent health concerns described with infectious diseases including dermatological conditions, otitis media, rheumatic heart disease and bronchectisis reported as common for very remote communities.

“Chronic disease is a huge concern; being isolated from specialists is a huge health concern. We have experienced mental health concerns with drought, floods and purely isolation as well.”

*(P8—Podiatrist)*

Participants were also asked if there is a need for community pharmacies to provide expanded services to address gaps in health service delivery. Responses for this comment varied with some participants describing well-serviced communities without the need for pharmacies to expand service delivery and those who described very limited access to health services and the potential expectation that pharmacies could address many gaps in health service delivery.

“I think it’s a great idea. In rural and remote areas, we don’t have enough skills; we don’t have the people available so I think that any one of us that is a health professional with a little bit of a broadened scope of practice does help out the clients but also the other health professionals within the area.”

*(P8—Podiatrist)*

Health professional perspectives of the expanded role of the pharmacist are influenced by political context, professional experiences and personal interactions with the pharmacy profession. Five major themes based on the role theory framework described the perspectives of the health professionals. Table 2 provides definitions of the role constructs and examples from the data [23].

### 3.1. Role Ambiguity

Allied health participants described various levels of uncertainty about a pharmacist’s role compared to doctors who felt they had a better understanding of the pharmacist’s traditional role. Inadequate knowledge about pharmacist training and even what tasks were considered standard practice for a pharmacist was commonly reported.

“It would be a benefit for services like remote nurses to understand what is in the pharmacy scope of practice and what you can offer. I don’t really know…. I just make ad hoc use of what I can, but I don’t really know what you can actually offer and do.”

*(P7—Remote Nurse Practitioner)*

This lack of clarity was also found for pharmacists’ understanding of other health professionals.

“Having a chat [with] our local pharmacist about what it is that we do, she was like, ‘Wow, I didn’t realise how broad it was,’ and that’s really important for us to know because, quite often, pharmacists are the front base in terms of medicine and access to healthcare that clients go to, and for her to advocate who she needs to see for whatever it is they are presenting with is important. The pharmacist here seems to have a limited understanding of what the services are.”

*(P2—Occupational Therapist)*

The notion that the pharmacy profession operates independently of the medical and allied health groups was also noted. Some participants suggested expanded practice may better incorporate the pharmacy profession.

“Pharmacy has sort of sat in that awkward middle ground: Are you medical? Are you with the psychologists that don’t fit either? Are you actually allied health? Where do you fit in allied health? I think you’ve always been sort of floating. Doing the expanded services is either going to drag you more into the medical field with the nurses or more towards allied health. You might get adopted somewhere.”

*(P3—Physiotherapist)*

### 3.2. Role Conflict

Professional conflict between doctors and pharmacists was most frequently described, and the expectation that pharmacists would be taking business away from other health providers by providing expanded services was also discussed. However, it was recognised that, for some expanded services, information and education is freely available and often not of high quality, and consequently, evidence-based pharmacy services would be an improvement on what is currently provided.

“The other barrier would be the other health professionals in the area: the doctors might be saying ‘You’re taking bread from my table, or you’re not qualified to provide that service and those sorts of issues’, but I think that depends on the services within the town. If the town is strapped for services, anyone with a bit of health knowledge is good…. People are getting dietary advice from their next-door neighbours, and that doesn’t put the dieticians out of business.”

*(P6—General Practitioner)*

Concerns were raised about community pharmacists not being able to separate their business role from their professional services role. Expectation that pharmacists would not be able to provide an entirely professional service due to selling of high sugar foods and products with no evidence-base in conjunction with evidence-based pharmaceuticals was described.

“Pharmacists could even change some of the things they have in their shops. They dabble in a bit of Darrell Lea [chocolate] stands, placement of that’s really important if it’s not near the counter. They definitely could influence what people are buying.”

*(P1—Public Health Nutritionist)*

In contrast, there was recognition that pharmacists are already providing a professional service from within retail pharmacies.

“Sometimes, it’s hard to be the recommender and the provider of a particular treatment…. The conflict of interest would be an interesting thing for pharmacists. But I suppose for pharmacies, people are always coming in saying they want something for something and pharmacists are always recommending things from their shelves.”

*(P6—General Practitioner)*

General practitioners also raised issues about pharmacists adopting a prescribing role with concern about the extent of training required to prescribe appropriately and quality control processes needed for safe prescribing.

“I don’t know how long pharmacists are going to expect to do extra training for. It’s certainly not going to be years unless they all change their profession. So, that’s my issue with prescribing, is that we have to know what we’re prescribing for.”

*(P5—General Practitioner)*

“If [pharmacists] are doing the prescribing, whose checking on them to make sure they are getting it right?”

*(P10—General Practitioner)*

Many other health professionals were supportive of expanding the scope of practice but agreed that structure and collaboration were needed to ensure clarity and acceptance about pharmacists’ expanded role.

“Have a conversation and collaborate, approach it cooperatively and have a partnership particularly with the GPs (general practitioners). We have been good buddies for a long time back. That sort of partnership can work really well.”

*(P6—General Practitioner)*

Comments were made about other professions including nurses who have adopted expanded practice and the issues they face with role conflict. Other health professionals, particularly doctors, were reported to be defensive about nurses and nurse practitioners expanding into their professional practice areas, and the issues that can arise for this role conflict were expected to be experienced by pharmacists.

“‘You’re encroaching!’ might come from the medical side. I know as nurses we do get that from certain avenues if they think that we are stealing their work.”

*(P19—Registered Nurse)*

“There will be things to manage about push back from other disciplines and other areas because people in health do get very protective of their patch.”

*(P7—Remote Nurse Practitioner/Midwife)*

In response, it was proposed that practitioners who might be resistant to expanded services and who are not working remotely need to re-evaluate who they expect to provide the health service role if they themselves are not willing to work in small communities.

“If they are really worried about it treading on their toes, why aren’t they out in community doing more? Why aren’t they going to the people? What’s their reason for not coming to the people? That’s where the problem is out there. People don’t want to come to them. They don’t want to leave their home. They have to cater for them at some point, you know, go to the communities to work.”

*(P22—Indigenous Health Worker)* (Context: doctor resistance)

### 3.3. Role Overload

Participants expressed concern about overload and that the pharmacist would be stretched too thin to be able to provide quality service. Inadequate resources including a shortage of pharmacists’ time and the lack of availability of pharmacists in the rural workforce was also noted.

“Can I expect someone to step into that role and be like, ‘Yeah, let’s give this a go,’ when you’re expanding your practice in many different areas at the same time, while you’re trying to learn everything at once? How can you learn everything very well if you’re putting that much pressure on yourself?”

*(P3—Physiotherapist)*

Some doctors saw the usual role of the pharmacist as sufficient and, thus, felt expanded practice was an unnecessary burden for the pharmacist.

“The pharmacist role is enough by itself without training for expanded practice.”

*(P5—General Practitioner)*

It was also suggested that an extension of the pharmacist role may be detrimental to the rural pharmacy workforce in that the expected overload would deter pharmacists from rural practice.

“They burn out and they don’t want to be a pharmacist anymore, and they go back to the city and do something else.”

*(P15—Occupational Therapist)*

### 3.4. Role Identity

The importance of role identity was discussed by participants describing the value in pharmacists reflecting on what their core business is and how expanded practice models may detract from this core business.

“You would need to be really careful about how you define expanded practice and how you link that to what pharmacy is and what pharmacists do. What it’s all about? What does it mean? Why am I a pharmacist if I am doing what a nurse is doing? Particularly for newer graduates, lack of professional direction, you can diversify so much that you take away from what is core....”

*(P15—Occupational Therapist)*

An individual approach to expanding the pharmacist’s role was also discussed in the context of each pharmacist having a different level of training, experience and interest in providing expanded practice based on their experience within their rural community.

“It is very pharmacist dependent because it is based on the extra skills that they may not have learnt, so that’s the good thing about remote pharmacists compared to city pharmacists.”

*(P4—Emergency Retrieval Doctor)*

Consideration of a specific title or accreditation for pharmacists who are trained to provide expanded services was suggested to aid recognition by other health professionals and the community. Similarly, a separation of community pharmacists from those providing professional clinical services was suggested.

“There should be two sorts of pharmacists: those that are paid a salary, work remote and go by the evidence, and they need to be separated from retail people who have a pharmacy degree and sell rubbish to patients who can’t afford it.”

*(P4—Emergency Retrieval Doctor)*

### 3.5. Role Insufficiency

Participants expressed concern about pharmacists not managing to undertake their usual medication management tasks effectively and, consequently, not being able to assume an expanded practice role. It was also noted that pharmacists have various skills related to medication management that are not being used effectively.

“It’s a shame because we are not using pharmacists enough in the role that they have got.”

*(P13—General Practitioner)*

Other health professionals including allied health and nurses were suggested as more suitable to perform an expanded role.

“I don’t think pharmacists can step into that role. If anyone can step into that role, it should be a nurse practitioner.”

*(P9—General Practitioner)*

In contrast, some participants felt the skills required to provide some expanded services included, for example, patient counselling that pharmacists are already well trained to provide, therefore not requiring further education.

“Low-level counselling type service is a service gap too. We have plenty of psychologists, but the sort of services that the minister used to provide or the trusted friend used to provide, those sorts of services, would be ok.”

*(P6—General Practitioner)*

In addition, although it was recognised that pharmacists may not currently have the skills required to provide expanded services, with appropriate training, they may be upskilled to be competent to practice within an expanded scope.

“Irrespective of your background, you need to be trained to do that. A doctor wouldn’t do a hearing test unless they were trained to do it, so if they do the same training as everyone else, I would have no problem with pharmacists doing it.”

*(P4—General Practitioner—Emergency Retrieval)*

A solution to the anticipated role insufficiency is adopting a collaborative and integrated approach, whereby pharmacists would perform expanded roles within a multidisciplinary team. It is expected that a collaborative model would raise the profile of the pharmacy profession and increase the acceptance of expanded pharmacy practice.

“It might actually help if they built their structures to actually link in more broadly with the other health services to link and to raise awareness of the role of how the pharmacist can help.”

*(P7—Remote Nurse Practitioner)*

## 4. Discussion

Appreciating the dynamics and interactions of key individuals in expanded models of care is important in developing frameworks, roles and scope of practice [17]. The necessity to set new practice standards and to establish cooperative relationships with other healthcare professionals is not new for pharmacy and was identified in 1990 by Hepler and Strand with the introduction of “pharmaceutical care” [3].

Rural pharmacists in Australia are widely recognised as the most accessible healthcare provider with the potential to deliver a much greater role in the health system [8]. This is common across many countries including Canada and New Zealand that share similar rural issues including a lack of access to health professionals working across vast distances and the consequent emergence of health professionals working to their full scope of practice [10]. The development of expanded services provided by rural pharmacists internationally is demonstrating small but positive impacts on health outcomes in rural and remote communities [10,14].

Pharmacists’ traditional role has been predominately to dispense medicines coupled with clinical review of prescriptions, labelling and professional activities such as medicine counselling to that ensure medicines are used safely and effectively [8]. As the scope of the pharmacist continues to evolve, it is important to understand the role theory that underpins other health professionals’ perspectives of an expanded role for rural community pharmacists.

A lack of understanding of health professionals’ roles and competencies has been recognised as a major barrier to effective interprofessional collaboration in primary healthcare provision [24]. This role ambiguity is also closely tied to role identity, which is highly subjective and based on an individual’s (pharmacist’s) interpretation of role expectation [17]. Both role ambiguity and identity were frequently identified in this study, with many participants being unsure of the boundary between pharmacists’ standard role compared to what would be considered an expanded role and how this expansion of practice would affect the pharmacist’s professional identity. Some participants were also uncertain of pharmacists’ standard role, and this highlighted the lack of integration of the pharmacist in collaborative care. The lack of clarity about projected roles and expectations, indicative of role ambiguity [17], is also an issue for pharmacists internationally and particular Australia, which has not yet developed a clear framework for expanded practice and consequently terms including extended, expanded and advanced are often used interchangeably, affording confusion [13]. This is despite peak professional pharmacy bodies in calling for a formal and system-wide recognition of advanced levels of practice and better support for expanded roles in isolated practice settings by 2023 [25]. Role ambiguity is thus detrimental to the advancement of the pharmacy profession and its ability to collaborate with other professionals. An approach to expanding pharmacy practice, where role identity and role ambiguity is addressed from the outset, would greatly assist pharmacists and other health professionals in connecting with and understanding pharmacists’ professional identity and thus in improving professional practice and collaboration. Consequently, it is recommended that pharmacy associations both within Australia and internationally develop a clear and concise definition of expanded practice that can be understood by all contributors to healthcare and the community.

Role conflict can occur when role occupants who have specific role expectations or certain behaviours associated with them are combined but incompatible [17]. Consequently, one person or group is unable to meet the expectation of another [17]. Nursing participants described instances where they regularly experienced role conflict with other health professionals, particularly the medical profession, with respect to their expanded scope of practice, and therefore, it is likely that pharmacists would experience similar tension. This was demonstrated in this study by a lack of support from GPs for pharmacists to undertake prescribing roles due to inexperience and limited training. In addition, concerns about pharmacists separating their retail businesses from professional practice were raised by some participants. These contributing factors to role conflict align with role boundary issues, scope of practice and accountability [26]. Doctors have also described concerns about accountability of the pharmacy profession, describing themselves as being ultimately accountable for patient care [26]. This notion was not shared by other professionals when exploring interprofessional conflict [26], with non-GP health professionals seeing themselves as each being accountable for their own work [26]. Any new professional practice model will be required to have a clear delineation of accountability and an expanded scope of practice for the pharmacist is no different.

Pharmacists have themselves raised concerns about their ability to fulfil role expectations in relation to expanded practice. Role insufficiency issues including touching patients, administering injections, making decisions and taking responsibility for decision making were discussed as challenges to providing expanded practice [27]. The question of “what is a pharmacist” and concern about readiness and ability to assume expanded practice tasks in Canada were identified, and this provides insight into whether pharmacists feel they have the skills and knowledge to expand practice [27]. In Australia, rural pharmacists do not appear to share the same concern. Evidence from a national study found that 91% of rural and remote pharmacists surveyed felt they had the skills and knowledge to implement expanded practice [28]. Regardless of pharmacists’ general feeling of competency, any expansion of practice should consider the availability of training to support pharmacists to ensure the provision of quality care for consumers. This notion has been cemented by a review of the literature reporting that pharmacists exhibit attitudes and attributes which favour their involvement in a wide range of healthcare services including expanded practice [25]. Ongoing systemic change is however required to facilitate system-wide extensions of pharmacists’ scope of practice [25].

Role overload can occur in rural and remote healthcare workers when the demands of a particular role exceed the individual person’s capacity to perform that role [17]. This lack of capacity is usually due to limitations in time, skill level and education or to complexity of the task [17]. Participants in this study described role overload as likely to occur in expanded practice for rural pharmacists due to limited staff and time available in an already highly occupied professional group. This concern was also shared by rural pharmacists in a study that identified barriers to expanded practice including time and space in addition to the longstanding concern of rural pharmacist workforce shortage [28]. Internationally, Canadian pharmacists expressed a desire for detailed instructions in terms of how to implement an expanded scope of practice, and this may reduce the impact of role overload [29]. This raises an important consideration for the feasibility of an expanded role for rural pharmacists in light of current rural workforce shortages.

## 5. Limitations

Years of pharmacy experience were not collected and have been inferred with complete accuracy from the age of the participants. However, the proportion of mature age pharmacy students in Australia is assumed to be low. Volunteer selection bias may be present in this study, and as such, it is not possible to ensure that all health professional perspectives are presented. Data saturation may have been achieved prematurely as a result of volunteer selection bias, whereby those with strong opinions, whether positive or negative, were more motivated to participate. However, a strength of the study is that it did include a range of health professionals from various locations to provide rigor and limit bias.

## 6. Conclusions

The evolving role of the rural pharmacist and the issues associated with the development of expanded scope of practice can be related to role theory. The lack of a clear boundary for the role of the pharmacist and consequent role ambiguity are major contributors to both role and interprofessional conflict. The development of a clear scope of practice including role delineation and competencies would potentially reduce this role conflict. In addition, the development of a system-wide acknowledgement of an expanded scope will also allow pharmacists and other health professionals to better understand pharmacists’ professional identity in the context of expanded practice. Shared and clarified accountability for pharmacists working in an expanded role are also likely to reduce role conflict, particularly with medical doctors. Ensuring that pharmacists providing expanded practice services are well resourced, trained and confident is also expected to limit problems with role overload and role insufficiency. This study has identified the many aspects of role theory that need to be carefully considered and worked through to ensure that the expanded scope of the pharmacy profession is smoothly implemented in Australia and widely accepted by pharmacists, health professionals and the community.

## Figures and Tables

**Table 1:** Demographic data for participants (N = 23).

Participant Characteristics	Number (%)
Age	
<25 years	2 (9%)
25–45 years	12 (52%)
46+ years	9 (39%)
Gender	
Female	18 (78%)
Male	5 (22%)
Occupation	
Doctor	8 (35%)
Nurse	4 (17%)
Allied Health	11 (48%)
MMM Categories	
MM3/4/5—Large/Medium/Large Rural Towns	5 (21%)
MM6—Remote Communities	16 (70%)
MM7—Very Remote Communities	2 (9%)

**Table 2:** Definition of role constructs and Examples [22].

Role Construct	Definition	Example
Role ambiguity	Disagreement on the role expectation associated with a lack of clarity of those expectations	*“A lot of the time, we just don’t know who to go to or who to refer to, and that’s both from the health professionals and a pharmacist: just not having a sound understanding of who provides what service.” (P2—Occupational Therapist)*
Role conflict	The focal person perceives existing role expectations as being contradictory or mutually exclusive	*“Pharmacists want to sell something to make money, and when they’re going to be providing the diagnostic and other services, then the incentive is going to be overwhelming.” (P5—General Practitioner)*
Role overload	Inadequate resources relative to the possibility of excessive demands	*“Time, not having enough time to do all of those things because you are busy doing other things…. You would have to really try and figure out your niche and what you wanted to do or provide, then focus on that level rather than broadening your scope the whole way.” (P8—Integrated Allied Health Manager-Podiatrist)*
Role identity	The individual’s interpretation of role expectation, that is, position-specific norms identifying the attitudes, behaviours and cognitions required and anticipated for a role occupant.	*“When you are doing lots of different things, you’re like, ‘Hang on a minute, how does this even relate to you?’… You need to have that capacity to bring it back to some core set of beliefs and structure around your professional identity.” (P15—Occupational Therapist)*
Role insufficiency	Disparity in fulfilling role expectations, obligations or goals as perceived by self or significant others	*“I’ve been to the chemist and never have seen the pharmacist. Some girl comes out and just gives you the pills. You know, where’s that comprehensive consult, or discussion about new medication? It’s not there.” (P17—General Practitioner)*

## Appendix A

Interview Questions for Semi-Structured Interview with Health Professionals.

1. Introduction—we are interested in hearing about your thoughts about expanded pharmacy services in rural and remote community pharmacies. Expanded pharmacy services are an extension of the recognised scope of the pharmacy profession. It is applied to pharmacists undertaking tasks usually provided by other health professionals, e.g., doctors, nurses and allied health, and is often synonymous and interchanged with the term extended practice.
2. Demographics
  (1) What is your age in complete years? _______	(2) What is your gender? ☐ Male ☐ Female ☐ Other, please specify ____________	(3) What is your home postcode? ______________	(4) What is your occupation? __________________
3. In your opinion, what are the major health concerns for your rural community?
4. What are your thoughts about rural community pharmacies providing expanded services?
5. Is there a need for community pharmacies to provide expanded pharmacy services in your community? Can pharmacies address any gaps in health service delivery?
6. Can you describe any expanded pharmacy services that would benefit your community? (interviewer to describe 5–10 expanded services for consideration if none are known)
7. What difficulties/barriers do you think might need to be overcome to implement expanded services?
8. What aspects/enablers would make expanded pharmacy services successful for your community?
9. What are your thoughts on remuneration for pharmacies providing services?
10. What impact do you think expanded services might have on health professional–pharmacist relationships?
11. Are there any other comments about expanded pharmacy practice you would like to make before we finish?
